# A Multidimensional Matrix Completion Method for 2-D DOA Estimation with L-Shaped Array

**DOI:** 10.3390/s25175583

**Published:** 2025-09-07

**Authors:** Haoyue Zhang, Junpeng Shi, Zhihui Li, Shuyun Shi

**Affiliations:** College of Electronic Countermeasure, National University of Defense Technology, Hefei 230037, China; zhanghaoyue23@163.com (H.Z.); lizhihui_16@163.com (Z.L.); 13478699873@163.com (S.S.)

**Keywords:** L-shaped array, 2-D DOA estimation, multidimensional matrix completion, sparse covariance fitting

## Abstract

This paper focuses on two-dimensional (2-D) direction-of-arrival (DOA) estimation for an L-shaped array. While recent studies have explored sparse methods for this problem, most exploit only the cross-correlation matrix, neglecting self-correlation information and resulting accuracy degradation. We propose a multidimensional matrix completion method that employs joint sparsity and redundant correlation information embedded in the covariance matrix to reconstruct a structured matrix compactly coupling the two DOA parameters. A semidefinite program problem formulated via covariance fitting criteria is proved equivalent to the atomic norm minimization framework. The alternating direction method of multipliers is designed to reduce computational costs. Numerical results corroborate the analysis and demonstrate the superior estimation accuracy, identifiability, and resolution of the proposed method.

## 1. Introduction

Two-dimensional (2-D) direction-of-arrival (DOA) estimation is a fundamental problem in array signal processing, with applications in radar, sonar, and wireless communications [1,2,3,4,5,6]. Various array geometries have been exploited for 2-D DOA estimation, including the uniform rectangular arrays (URAs), the uniform circular arrays (UCAs), L-shaped arrays, etc. Compared to a URA or a UCA, an L-shaped array achieves satisfactory estimation performance with significantly fewer sensors, reducing hardware cost and computational complexity. Therefore, the 2-D DOA estimation for L-shaped arrays has attracted considerable attention.

Research on 2-D DOA estimation for L-shaped arrays originated with [7] and has since evolved through diverse approaches. Compared to 1-D DOA estimation, the increased dimensionality inherently imposes a higher computational burden [8]. Numerous methods have been proposed to mitigate this problem [9,10,11,12,13]. A category of approaches applies 1-D DOA estimation separately to each subarray followed by a pairing procedure [14], which may lead to performance degradation or failure. Subsequently, methods with automatic pairing were devised based on the cross-correlation matrix (CCM), such as joint singular value decomposition [15], a decoupled method based on Jacobi-Anger expansion [16] and parallel factor analysis [17]. Since most methods overlook the multidimensional signal structure, a tensor-based 2-D DOA estimation method [18] is developed, removing the cross-term generated from the correlated co-array signal and noise components with enhanced accuracy.

Although CCM-based methods reduce computational complexity, most face an inherent limitation that they cannot identify sources exceeding the number of sensors in each subarray. An aperture and snapshots extension technique [19] increased the maximum identifiable sources for L-shaped arrays to $M_{\mathrm{total}}-2$ (where $M_{\mathrm{total}}$ is the total number of sensors), yet only one subarray fully leveraged all data for improved accuracy. The authors [20] proposed a canonical polyadic decomposition-based approach to achieve approximately $0.34{\left(M_{\mathrm{total}}/2+1\right)}^{2}$ degrees of freedom, significantly improving the identifiability. Sparse arrays [21,22,23] are also designed as the subarrays of an L-shaped array to increase the degrees of freedom of the system.

Compressed sensing has spurred significant interest in sparse methods for DOA estimation [24,25,26,27], exhibiting enhanced robustness under limited snapshots or low signal-to-noise (SNR). These methods discretize the parameter domain into a finite grid, assuming targets lie on these points, framing DOA estimation as a sparse recovery problem [28,29]. The continuous nature of the domain causes grid mismatch, while excessively dense grids induce robustness issues due to high inter-atom correlation. Consequently, gridless methods operating in the continuous domain were developed to maintain high resolution under challenging conditions where subspace methods degrade [30,31]. Prominent gridless approaches include deterministic methods, such as atomic norm minimization (ANM) and its variants [32], and covariance fitting methods [33,34].

Although the ANM framework has been extended to multidimensional problems [35,36,37], these extensions are primarily designed for URA or MIMO systems with sufficient data. For L-shaped arrays, the parameters of two subarrays are completely decoupled in the covariance matrix. Most CCM-based sparse methods [21,37] exploit only cross-correlation information. Furthermore, vectorizing the covariance matrix [21] neglects the inherent joint sparsity across snapshots. Therefore, it is desirable to design a sparse method fully utilizing all available data to improve identifiability and accuracy.

In this paper, we propose a sparse covariance fitting method for 2-D DOA estimation using L-shaped arrays. Drawing on the connection between the multidimensional structured covariance matrix and the multilevel Toeplitz (MLT) structure [36], the method leverages the joint sparsity and redundant information across subarrays to recover the structured matrix and enhance estimation performance. The main contributions are summarized as follows:

- We propose a matrix completion method leveraging the intrinsic connection between the covariance matrix and MLT structure. It simultaneously captures the joint sparsity and the coherence between signals received by the two subarrays. An efficient alternating direction method of multipliers (ADMM) algorithm is further developed to reduce computational complexity. The proposed method extends the virtual aperture through an interpolation-like mechanism, thereby improving estimation accuracy and identifiability (see Section 3 and Section 4).
- Extensive simulation results demonstrate the superior performance of the proposed method in terms of identifiability and statistic estimation accuracy benchmarked by the Cramér-Rao bound (CRB). The computational efficiency gained by the ADMM implementation is also empirically verified (see Section 6).

*Notations*: Boldface lowercase/uppercase letters denote vectors/matrices. R and C represent the sets of real and complex numbers. ${\left(\cdot \right)}^{T}$, ${\left(\cdot \right)}^{*}$, ${\left(\cdot \right)}^{H}$ denote the transpose, complex conjugate, conjugate transpose operator. ${∥\cdot ∥}_{2}$, ${∥\cdot ∥}_{F}$ denote the $\ell_{2}$ norm and Frobenius norm. Key matrix operations comprise the trace tr(·), rank rank(·), diagonal matrix formation diag(·), and the real-valued inner product ${\langle X,Y\rangle }_{R}=ℜ\left\{\mathrm{tr}\left(X^{H}Y\right)\right\}$. Matrix indexing uses $X_{i,:}/X\left(i,:\right)$ for the *i*-th row, $X_{:,j}/X\left(:,j\right)$ for the *j*-th column, and $X_{i,j}$ for the (i,j)-th element. ⊗,⊙,∘ denote the Kronecker, Hadamard and Khatri-Rao product. $I_{n}$ is an n×n identity matrix and X⪰0 denotes a Hermitian positive semidefinite (PSD) matrix. ℜ{·} and ℑ{·} denote the real and imaginary part of a complex number.

## 2. Preliminaries

### 2.1. Basic Model

**Figure 1:**
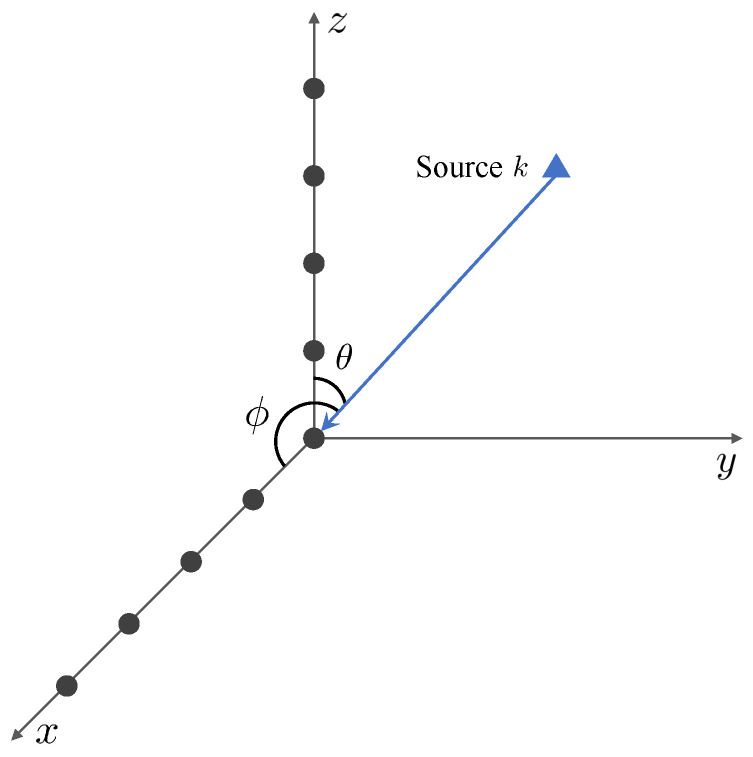
The schematic diagram of an L-shaped array.

We consider an L-shaped array configured with two orthogonal ULAs with $M_{x}$ and $M_{z}$ sensors at x-axis and z-axis, respectively. The spacing of sensors is assumed to be half of the wavelength and *K* uncorrelated narrowband sources impinge on the array with azimuth angles $\phi =\{\phi_{1},\ldots ,\phi_{K}\}$ and elevation angles $\theta =\{\theta_{1},\ldots ,\theta_{K}\}$ as Figure 1. The received signal with *L* snapshots can be represented as follows:

(1) $$\begin{array}{cc} X & =\sum_{k=1}^{K}a_{x}\left(\phi_{k}\right)s_{k}+E_{x}=A_{x}\left(\phi \right)S+E_{x},\\ Z & =\sum_{k=1}^{K}a_{z}\left(\theta_{k}\right)s_{k}+E_{z}=A_{z}\left(\theta \right)S+E_{z}, \end{array}$$

where $A_{x}\left(\phi \right)=\left[a_{x}\left(\phi_{1}\right),\ldots ,a_{x}\left(\phi_{K}\right)\right]$ and $A_{z}\left(\theta \right)=\left[a_{z}\left(\theta_{1}\right),\ldots ,a_{z}\left(\theta_{K}\right)\right]$ are manifold matrices of subarrays, $a_{x}\left(\phi_{k}\right)={\left[1,e^{i\pi \cos\phi_{k}},\ldots ,e^{i\pi \left(M_{x}-1\right)\cos\phi_{k}}\right]}^{T}\in C^{M_{x}}$ and $a_{z}\left(\theta_{k}\right)={\left[e^{i\pi \cos\theta_{k}},\ldots ,e^{i\pi \left(M_{z}-1\right)\cos\theta_{k}}\right]}^{T}\in C^{M_{z}-1}$; $S={\left[s_{1},\ldots ,s_{K}\right]}^{T}$ denote the uncorrelated source signals; $E={\left[E_{x}^{T},E_{z}^{T}\right]}^{T}$ is the spatially and temporally independent Gaussian white noise with variance $\sigma^{2}$. The signals are concatenated as follows:

(2) $$\begin{array}{c} Y=\left[\begin{array}{c} A_{x}\left(\phi \right)\\ A_{z}\left(\theta \right) \end{array}\right]S+E\in C^{M\times L}, \end{array}$$

where $M\;=\;M_{x}+M_{z}-1$ is the total number of sensors. For notational simplicity, we will write $A_{x}\left(\phi \right),A_{z}\left(\theta \right),a_{x}\left(\phi_{k}\right),a_{z}\left(\theta_{k}\right)$ as $A_{x},A_{z},a_{xk},a_{zk}$ hereafter.

The covariance matrix of Y is denoted as follows:

(3) $$\hat{R}=\left[\begin{array}{c} A_{x}\\ A_{z} \end{array}\right]P{\left[\begin{array}{c} A_{x}\\ A_{z} \end{array}\right]}^{H}+\sigma^{2}I_{M}=\left[\begin{array}{cc} R_{x} & R_{xz}\\ R_{xz}^{H} & R_{z} \end{array}\right]+\sigma^{2}I_{M}=R+\sigma^{2}I_{M},$$

where $P=\mathrm{diag}(p_{1},\cdots ,p_{K})$ and $p_{k}>0$ represents the power of the *k*-th source. $R_{x}$, $R_{z}$ are noise-free self-correlation matrices of X and Z, and $R_{xz}$ is the cross-correlation matrix between X and Z. For the case of infinite snapshots, the sample covariance matrix can be calculated as follows:

(4) $$\begin{array}{c} \tilde{R}=\frac{1}{L}YY^{H}. \end{array}$$

### 2.2. Joint Sparse Recovery Framework

The signals sampled at two subarrays share identical complex amplitude S, and we can exploit their correlation to define the atomic set as follows [38]:

(5) $$\begin{array}{c} A:=\{a\left(\phi ,\theta ,\psi \right)=\left[\begin{array}{c} a_{x}\left(\phi \right)\\ a_{z}\left(\theta \right) \end{array}\right]\psi =a\left(\phi ,\theta \right)\psi \} \end{array}$$

where $\psi \in C^{1\times L}\left({∥\psi ∥}_{2}=1\right)$ embeds the joint sparsity across snapshots and a(ϕ,θ) couples parameters of two subarrays. The joint sparse recovery (JSR) framework is formulated based on a 2-D overcomplete dictionary composed of atoms in the set as follows:

(6) $$\begin{array}{cc} \; & \min_{S}\frac{1}{2}∥Y-A_{0}{S∥}_{F}^{2}+\lambda {∥S∥}_{2,1}, \end{array}$$

where $A_{0}={\left[\begin{array}{c} A_{x}{\left(\phi_{0}\right)}^{T},A_{z}{\left(\theta_{0}\right)}^{T} \end{array}\right]}^{T}$ is the dictionary matrix composed of $N_{0}$ candidate atoms with the regularization parameter λ set as [39], $\theta_{0}$ and $\phi_{0}$ denote the azimuth and elevation angles of candidate atoms in the dictionary. The former item measures the data fidelity and correlation while the latter item constrains the joint sparsity via the $\ell_{2,1}$ norm. Although the framework exploits inter-signal correlation and joint sparsity to enhance estimation performance, it exhibits all characteristic limitations of grid-based sparse methods [38].

## 3. Multidimensional Matrix Completion Method

It is intuitive to formulate the covariance fitting criteria for $\hat{R}$ in (3) from the generalized least squares framework [29,40], written as follows:

(7) $$\begin{array}{c} h_{1}=\left\{\begin{array}{cc} ∥{\hat{R}}^{-\frac{1}{2}}\left(\tilde{R}-\hat{R}\right){\tilde{R}}^{-\frac{1}{2}}{∥}_{F}^{2}, & L\geq M\\ ∥{\hat{R}}^{-\frac{1}{2}}\left(\tilde{R}-\hat{R}\right){∥}_{F}^{2}, & L<M \end{array}\right.. \end{array}$$

Considering the case of L≥M, the objective function can be simplified as follows:

(8) $$\begin{array}{cc} h_{1} & =\mathrm{tr}[\left(I_{M}-{\tilde{R}}^{-1}\hat{R}\right)\left({\hat{R}}^{-1}\tilde{R}-I_{M}\right)]\\ \; & =\mathrm{tr}\left({\tilde{R}}^{-1}\hat{R}\right)+\mathrm{tr}\left({\tilde{R}}^{\frac{1}{2}}{\hat{R}}^{-1}{\tilde{R}}^{\frac{1}{2}}\right)-2M. \end{array}$$

The MLT structure [36] enables reparameterizing $\hat{R}$ as a linear function of the DOAs. This parameterization relies fundamentally on the Vandermonde decomposition theorem for a MLT matrix as follows:

**Theorem** **1** ([36])**.** *A PSD N-level Toeplitz matrix $T_{N}$ can be decomposed as follows:*

(9) $$\begin{array}{c} T_{N}={\hat{A}}_{N}P{\hat{A}}_{N}^{H},\;\;{\hat{A}}_{N}=A_{N}\left(f_{N}\right)\circ \cdots \circ A_{1}\left(f_{1}\right), \end{array}$$

*where $P=\mathrm{diag}(p_{1},\ldots ,p_{r})$, $p_{i}>0$ and $\mathrm{rank}(T_{N})=r$. The manifold matrices ${\left\{A_{i}\left(f_{i}\right)\right\}}_{i=1}^{N}$ have Vandermonde structure as $A_{x}\left(\phi \right)$ with distinct $f_{i}={\left\{f_{ik}\right\}}_{k=1}^{r}$ corresponding to ${\left\{\cos\phi_{k}\right\}}_{k=1}^{K}$ in the steering vector. The sufficient condition to guarantee the uniqueness of decomposition is $r<\min{\left\{M_{n}\right\}}_{n=1}^{N}$.*

The relationship between $\hat{R}$ and an MLT matrix is established as follows:

**Proposition** **1.** *Assuming the matrix $T_{N}$ has unique Vandermode decomposition as (9), the following holds:*

(10) $$\begin{array}{cc} R_{mn} & =A_{m}\left(f_{m}\right)PA_{n}^{H}\left(f_{n}\right)=T_{N}\left(1:N_{m-1}:N_{m},1:N_{n-1}:N_{n}\right), \end{array}$$

*where $N_{q}=\left\{\begin{array}{cc} \prod_{p=1}^{q}M_{p}, & q\geq 1\\ 1, & q=0 \end{array}\right.$.*

**Proof.**  See Appendix A.    □

The ideal covariance matrix R in (3) can be reparameterized from a two-level MLT matrix $T_{2}\in C^{M_{1}M_{2}\times M_{1}M_{2}}$ with $M_{1}=M_{x},M_{2}=M_{z}$ as follows:

(11) $$\begin{array}{cc} R_{x} & =A_{x}PA_{x}^{H}=T_{2}\left(1:M_{1},1:M_{1}\right)=A_{1}PA_{1}^{H},\\ R_{xz} & =A_{x}PA_{z}^{H}=T_{2}\left(1:M_{1},M_{1}+1:M_{1}:M_{1}M_{2}\right)=A_{1}PA_{2}{\left(2:M_{2},:\right)}^{H},\\ R_{z} & =A_{z}PA_{z}^{H}=T_{2}\left(M_{1}+1:M_{1}:M_{1}M_{2},M_{1}+1:M_{1}:M_{1}M_{2}\right)\\ & =A_{2}\left(2:M_{2},:\right)PA_{2}{\left(2:M_{2},:\right)}^{H}. \end{array}$$

Then function in (8) is formulated as a semidefinite program (SDP) problem, written as follows:

(12) $$\begin{array}{cc} & \min_{X,T_{2},\sigma }\mathrm{tr}\left(X\right)+\mathrm{tr}\left({\tilde{R}}^{-1}\hat{R}\right)\\ \; & s.t.\left[\begin{array}{cc} X & {\tilde{R}}^{\frac{1}{2}}\\ {\tilde{R}}^{\frac{1}{2}} & \hat{R} \end{array}\right]⪰0,T_{2}\;⪰\;0,\mathrm{rank}\left(T_{2}\right)\;\leq \;\min{\left\{M_{n}\right\}}_{n=1}^{2}-1, \end{array}$$

where the rank constraint is non-convex and limits identifiable targets. Therefore, we consider relaxing the rank constraint and derive the optimal solution $T_{2}^{*}$ and $\sigma^{*}$. Similar to SPA in [33], there exists one redundant variable at the principal diagonal of $\hat{R}$, indicating $T_{2}^{*}$ can be represented as follows:

(13) $$\begin{array}{c} T_{2}^{*}=\left(A_{2}\circ A_{1}\right)P{\left(A_{2}\circ A_{1}\right)}^{H}+\delta I, \end{array}$$

where $\delta =\sigma^{2}-\sigma^{*2}$ is the redundant variable. It follows that $\mathrm{rank}(T_{2}^{*})\geq K$ and the solution of (12) is a special realization with δ=0. It is imperative to perform post-processing on $T_{2}^{*}$, typically via *K*-order truncated eigendecomposition, to retain only the signal subspace. Furthermore, the classical multidimensional ESPRIT (MD-ESPRIT) algorithm can be applied for 2-D angles extraction and auto-pairing [41].

The shared amplitude S establishes a bridge from the covariance matrix into a structured matrix with compactly coupled parameters. The optimization induces an interpolation-like effect, extending the virtual aperture to enhance identifiability. Crucially, the quantifiable identifiability of the proposed method is governed by the necessary and sufficient conditions for unique Vandermonde decomposition of $T_{2}$, which can be weaker than in Theorem 1 [36] with rigorous verification remaining an open problem. Therefore, we have not theoretically verified the maximum identifiable sources, but we will demonstrate its superiority over existing methods through simulations in Section 6.1.

**Remark** **1.** *Similar to the GLS method in 1-D DOA estimation [42], the proposed method has close connection to the ANM framework as follows:*

**Corollary** **1.** *The SDP problem in (12), after rank constraint relaxation, is equivalent to the following reweighted ANM (RAM) [43] formulation as follows:*

(14) $$\begin{array}{c} \min_{Z}\sqrt{M}{∥Z∥}_{A^{w}}+\sum_{i=1}^{M}\sqrt{{\left({\tilde{R}}^{-1}\right)}_{i,i}}{∥{\left({\tilde{R}}^{\frac{1}{2}}-Z\right)}_{i,:}∥}_{2}, \end{array}$$

*where ${∥Z∥}_{A^{w}}$ denotes the weighted atomic norm defined as follows:*

(15) $$\begin{array}{c} {∥Z∥}_{A^{w}}:=\underset{\phi ,\theta ,s_{k}}{\inf}\left\{\sum_{k=1}^{K}\frac{∥s_{k}{∥}_{2}}{w_{k}}:Z=\sum_{k=1}^{K}a\left(\phi_{k},\theta_{k}\right)s_{k}\right\}, \end{array}$$

*with*

(16) $$\begin{array}{c} w_{k}={\left[\frac{1}{M}a{\left(\phi_{k},\theta_{k}\right)}^{H}Wa\left(\phi_{k},\theta_{k}\right)\right]}^{-\frac{1}{2}},W={\tilde{R}}^{-1}. \end{array}$$

*The problem can also be transformed to the joint sparse framework in (6) with $\hat{R}$ in (12) set as $R+\lambda I_{M}$ and the joint sparse metric substituted as follows:*

(17) $$\begin{array}{c} {∥S∥}_{2,w}=\sum_{i=1}^{N_{0}}\frac{∥s_{i}{∥}_{2}}{w_{i}}, \end{array}$$

*where $N_{0}$ denotes the number of selectable grid points in the 2-D overcomplete dictionary matrix and $w_{i}$ is defined in (16).*

*It follows that the joint sparsity is effectively exploited and the atom weight is capable of adjusting the influence of different sources.*

**Remark** **2.** *It is desirable to analyze the atom weight of the k-th target $w_{k}$ in (16) as it provides some insights into the proposed method.*

**Theorem** **2.** *Suppose that $W={\left(R+\epsilon I\right)}^{-1}$, which is the ideal form of sample covariance matrix, then $w_{k}$ in (16) can be calculated as follows:*

(18) $$\begin{array}{c} w_{k}=\sqrt{\epsilon }(1-\frac{1}{M}∥Q^{-\frac{1}{2}}\left(A_{x}^{H}a_{xk}+A_{z}^{H}a_{zk}\right){{∥}_{2}^{2})}^{-\frac{1}{2}}, \end{array}$$

*where $Q=\epsilon P^{-1}+MI_{K}$, $A={\left[A_{x}^{T},A_{z}^{T}\right]}^{T}$ is the manifold of R in W.*

**Proof.**  See Appendix B.    □

It follows that the atom weights actually depend on the correlation between the atoms to be chosen and all the atoms embedded in W, which means that the DOAs around the corresponding atoms in W are more likely to be selected. Besides, the weights of different sources are also influenced by Q embedding the power of sources. The analysis above shows that the proposed method is capable of utilizing the correlation of different subarrays by the weighted atom norm while allowing for the assignment of distinct weights to different atoms.

**Remark** **3.** *Suppose $P=2M_{x}M_{z}-M_{x}-M_{z}+1$ denotes the number of variables in $T_{2}$, the proposed method has the complexity of $O(n_{1}^{2}n_{2}^{2.5})$, where $n_{1}={\left(M_{x}+M_{z}-1\right)}^{2}+P$ and $n_{2}=M_{x}+M_{z}-1+M_{x}M_{z}$. It can be seen that the complexity of the proposed method only depends on the number of sensors in the array.*

## 4. Efficient Implementation via ADMM

Although the non-convex constraint can be tackled by post-processing, the reuse of elements in the MLT matrix $T_{2}$ still leads to high computational cost, which means that the general SDP3 solver is not suitable for practical use. ADMM [44] is an effective approach for reducing the computational complexity of SDP problems [24]. Therefore, we will apply it to the optimization model in (14) with the separable variables.

### 4.1. Algorithm Framework

The auxiliary variables $Q_{1}$, $Q_{2}$ are introduced to reformulate (14) as follows:

(19) $$\begin{array}{cc} \min_{X,Z,R,Q_{1},Q_{2}} & \frac{1}{2}\left[\mathrm{tr}\left(X\right)+\mathrm{tr}\left({\tilde{R}}^{-1}R\right)\right]+\delta_{S_{+}}\left(Q_{1}\right)+\delta_{S_{+}}\left(Q_{2}\right)\\ & +\sum_{i=1}^{M}\sqrt{{\left({\tilde{R}}^{-1}\right)}_{i,i}}{∥{\left({\tilde{R}}^{\frac{1}{2}}-Z\right)}_{i,:}∥}_{2}\\ & s.t.\;Q_{1}=\left[\begin{array}{cc} X & Z^{H}\\ Z & R \end{array}\right],Q_{2}=T_{2}, \end{array}$$

where $\delta_{S_{+}}\left(\cdot \right)$ is the indicator function of the PSD matrices set, i.e., $\delta_{S_{+}}\left(Q_{i}\right)=0$ if $Q_{i}⪰0,i=1,2$ or *∞* otherwise.

Assume the Toeplitz operator is defined as follows:

$$\begin{array}{c} T\left(u\right)=\left[\begin{array}{cccc} u_{0} & u_{1} & \cdots & u_{n-1}\\ u_{-1} & u_{0} & \cdots & u_{n-2}\\ \vdots & \vdots & \ddots & \vdots \\ u_{1-n} & u_{2-n} & \cdots & u_{0} \end{array}\right],u={\left[u_{1-n},\cdots ,u_{0},\cdots ,u_{n-1}\right]}^{T}\in C^{2n-1}, \end{array}$$

and $T_{2}$ is composed of the elements in a PSD matrix $T\in C^{M_{2}\times \left(2M_{1}-1\right)}$ as follows:

(20) $$\begin{array}{c} T_{2}\;=\;\left[\begin{array}{cccc} T(T_{1,:})\; & \;{\left(T\left(T_{2,:}\right)\right)}^{H}\; & \;\cdots \; & \;{\left(T\left(T_{M_{2},:}\right)\right)}^{H}\\ T(T_{2,:})\; & \;T(T_{1,:})\; & \;\cdots \; & \;{\left(T\left(T_{M_{2}-1,:}\right)\right)}^{H}\\ \vdots \; & \;\vdots \; & \;\ddots \; & \;\vdots \\ T(T_{M_{2},:})\; & \;T(T_{M_{2}-1,:})\; & \;\cdots \; & \;T(T_{1,:}) \end{array}\right], \end{array}$$

then R can be reparameterized by T according to (11) and (20).

It follows that the augmented Lagrangian function of (19) is given by the following:

(21) $$\begin{array}{cc} \; & L\left(Q,X,Z,T,\Lambda \right)=\frac{1}{2}\left[\mathrm{tr}\left(X\right)+\mathrm{tr}\left({\tilde{R}}^{-1}R\right)\right]+\sum_{i=1}^{M}\sqrt{{\left({\tilde{R}}^{-1}\right)}_{i,i}}{∥{\left({\tilde{R}}^{\frac{1}{2}}-Z\right)}_{i,:}∥}_{2}\\ & +\delta_{S_{+}}\left(Q_{1}\right)+\delta_{S_{+}}\left(Q_{2}\right)+\frac{\mu_{1}}{2}∥Q_{1}-\left[\begin{array}{cc} X & Z^{H}\\ Z & R \end{array}\right]+\mu_{1}^{-1}\Lambda_{1}{∥}_{F}^{2}-\frac{1}{2\mu_{1}}{∥\Lambda_{1}∥}_{F}^{2}\\ \; & +\frac{\mu_{2}}{2}∥Q_{2}-T_{2}+\mu_{2}^{-1}\Lambda_{2}{∥}_{F}^{2}-\frac{1}{2\mu_{2}}{∥\Lambda_{2}∥}_{F}^{2}, \end{array}$$

where $\mu_{1}$, $\mu_{2}$ are penalty parameters and $\Lambda_{1}$, $\Lambda_{2}$ are Lagrangian multipliers.

The ADMM is implemented by equating the derivative of the objective function in (21) with respect to each variable to be zero. Therefore, the algorithm iterates the following steps:   

(22) $$\begin{array}{cc} Q_{1}^{l+1}=P_{S_{+}}(\left[\begin{array}{cc} X^{l} & {\left(Z^{l}\right)}^{H}\\ Z^{l} & R^{l} \end{array}\right]\; & -\mu_{1}^{-1}\Lambda_{1}^{l}),\;Q_{2}^{l+1}=P_{S_{+}}\left(T_{2}^{l}-\mu_{2}^{-1}\Lambda_{2}^{l}\right),\\ \left(X^{l+1},Z^{l+1},T^{l+1}\right) & =\underset{X,Z,T}{\arg\min}L\left(Q_{1}^{l+1},Q_{2}^{l+1},X,Z,T,\Lambda_{1}^{l},\Lambda_{2}^{l}\right),\\ \Lambda_{1}^{l+1} & =\Lambda_{1}^{l}+\mu_{1}\left(Q_{1}^{l+1}-\left[\begin{array}{cc} X^{l+1} & {\left(Z^{l+1}\right)}^{H}\\ Z^{l+1} & R^{l+1} \end{array}\right]\right),\\ \Lambda_{2}^{l+1} & =\Lambda_{2}^{l}+\mu_{2}\left(Q_{2}^{l+1}-T_{2}^{l+1}\right), \end{array}$$

where $P_{S_{+}}\left(\cdot \right)$ is the projection onto the set of PSD matrices and it is implemented by eigendecomposition with only positive eigenvalues preserved. The iteration is terminated when primal residuals $\epsilon_{p}$ and dual residuals $\epsilon_{d}$ satisfy the stopping criteria [44], i.e., $\epsilon_{p}<\epsilon^{\mathrm{pri}},\epsilon_{d}<\epsilon^{\mathrm{dual}}$, or the number of iterations reaches a pre-defined limit. The closed-form solutions for X,Z,and T in (22) are provided in the next section.

### 4.2. Closed-Form Solution of Subproblem

Write the Hermitian matrix $Q_{1}$ as follows:

(23) $$\begin{array}{c} Q_{1}=\left[\begin{array}{cc} Q_{11} & Q_{21}^{H}\\ Q_{21} & Q_{22} \end{array}\right], \end{array}$$

and the matrix $\Lambda_{1}$ is partitioned similarly.

Since the variables X, Z, and T are separable, they can be separately calculated by solving subproblems in (22) with other variables fixed. Then we can get the solution in closed form via equating the derivative of the objective function with respect to {X,Z} to be zero as follows:

(24) $$\begin{array}{c} X^{l+1}=Q_{11}^{l+1}+\mu_{1}^{-1}\left(\Lambda_{11}^{l}-I_{M}/2\right), \end{array}$$

(25) $$\begin{array}{c} Z^{l+1}\;=\;{\left(\Sigma -\mu_{1}I\right)}^{-1}\left(\Sigma {\tilde{R}}^{\frac{1}{2}}-\mu_{1}Q_{21}^{l+1}-\Lambda_{21}^{l}\right), \end{array}$$

where Σ is a diagonal matrix with the *i*-th element as follows:

(26) $$\begin{array}{c} \eta_{i}=-\frac{\sqrt{{\left({\tilde{R}}^{-1}\right)}_{i,i}}}{2∥{\left({\tilde{R}}^{\frac{1}{2}}-Z\right)}_{i,:}{∥}_{2}}, \end{array}$$

and we may use $Z^{l}$ here as an approximation.

Since elements of T depend on both R and $T_{2}$, the subproblem is written as follows:

(27) $$\begin{array}{cc} T^{l+1} & =\underset{T}{\arg\min}\mathrm{tr}\left({\tilde{R}}^{-1}R\right)+\mu_{1}{∥Q_{22}^{l+1}-R+\mu_{1}^{-1}\Lambda_{22}^{l}∥}_{F}^{2}\\ & +\mu_{2}{∥Q_{2}^{l+1}-T_{2}+\mu_{2}^{-1}\Lambda_{2}^{l}∥}_{F}^{2}. \end{array}$$

The variables $u={\left[\begin{array}{c} u_{1}^{T},u_{2}^{T} \end{array}\right]}^{T}$ in R can be represented as follows:

(28) $$\begin{array}{cc} u_{1} & =R_{x}\left(:,1\right)={\left(T_{1,M_{1}:-1:1}\right)}^{T},\;u_{2}=\mathrm{vec}\left(R_{xz}^{H}\right)=\mathrm{vec}\left(T_{2:M_{2},M_{1}:2M_{1}-1}\right), \end{array}$$

and $R_{z}\left(:,1\right)={\left[u_{1}\left(1\right),u_{2}{\left(1:M_{2}-2\right)}^{T}\right]}^{T}$. Suppose R(·) is the adjoint operator with

(29) $$\begin{array}{c} \mathrm{tr}\left(GR\right)={\langle R\left(G\right),u\rangle }_{R}, \end{array}$$

where G is an M×M Hermitian matrix, then $g=R\left(G\right)={\left[\begin{array}{c} g_{1}^{T},g_{2}^{T} \end{array}\right]}^{T}$ can be calculated as follows:   

(30) $$\begin{array}{cc} g_{1}\left(l\right) & =\left\{\begin{array}{cc} \sum_{i=j=1}^{M_{1}+1}G_{i,j}, & l=1\\ \sum_{i-j=l-1}G_{i,j},\;\;i,j\leq M_{1}, & l\in [2,M_{1}] \end{array}\right.,\\ g_{2}\left(l\right) & =\left\{\begin{array}{cc} \sum_{i-j=l}G_{i,j}+G_{(M_{1}+l),1},i,j\geq M_{1}+2, & l\in [1,M_{2}-2]\\ g_{0}\left(l\right),\;\;g_{0}=\mathrm{vec}\left(G_{\left(M_{1}+1\right):M,1:M_{1}}\right), & l\geq M_{2}-1 \end{array}\right., \end{array}$$

Then we reconstruct g to the structure of T as $R_{T}\left(G\right)$ according to (28). We can similarly define the operator $T_{N}\left(\cdot \right)$ to calculate the sum of the same element in a two-level MLT matrix as (20) and reconstruct it to the structure of T. Subsequently, the closed-form solution can be obtained by letting the derivation of the function in (27) be zero as follows:

(31) $$\begin{array}{c} T^{l+1}=D^{\circ -1}⊙\left[R_{T}\left(R_{0}\right)+T_{N}\left(T_{0}\right)\right],\\ R_{0}=\mu_{1}Q_{22}^{l+1}\;+\;\Lambda_{22}^{l}\;-\;\frac{1}{2}{\tilde{R}}^{-1},\;T_{0}=\mu_{2}Q_{2}^{l+1}\;+\;\Lambda_{2}^{l}, \end{array}$$

where $D^{\circ -1}$ denotes the element-wise reciprocal matrix of D, $D=\mu_{1}D_{1}+\mu_{2}D_{2}$, and $D_{1}$, $D_{2}$ are count matrices which record the number of corresponding elements in $R_{0}$ and $T_{0}$ to construct T.

In summary, the proposed ADMM is presented in Algorithm 1.
**Algorithm 1** ADMM for Multidimensional Matrix Completion**Input:**
    $\tilde{R},\mu_{1},\mu_{2}$ and model order *K*
**Output:**
    θ,ϕ
    Initialize $X^{0}$,$Z^{0}$,$T^{0}$,$\Lambda_{1}^{0},\Lambda_{2}^{0},l=0$;
    **for** 
$l<l_{\max}$ 
**do**
        1: Update $Q_{1}^{l+1}$,$Q_{2}^{l+1}$ via (22);
        2: Update $X^{l+1}$ via (24);
        3: Update $Z^{l+1}$ via (25) with Σ set as (26);
        4: Calculate $R_{0}$ and $T_{0}$ via (31);
        5: Reconstruct $R_{T}\left(R_{0}\right)$ and $T_{N}\left(T_{0}\right)$ via (30) and (20);
        6: Update $T^{l+1}$ via (31);
        7: Update $T^{l+1}$ via (31);
        8: Update $\Lambda_{1}^{l+1}$,$\Lambda_{2}^{l+1}$ via (22);
        9: l←l+1;
        **if** $\epsilon_{p}\geq \epsilon^{\mathrm{pri}},\epsilon_{d}\geq \epsilon^{\mathrm{dual}}$ **then**
            break
        **end if**
**    end for**
    10: Reconstruct $T_{2}$ via T and postprocess $T_{2}$ with *K*-order truncated eigendecomposition to obtain $T_{2}^{K}$;
    11: Extract and pair the DOA groups using MD-ESPRIT algorithm;
    12: **return** θ,ϕ.


## 5. CRB

The CRB is a powerful benchmark to evaluate the performance of the paramter estimators. For general 1-D homogeneous noise case, according to the research in [45], the CRB can be calculated as follows:

(32) $$\begin{array}{c} \mathrm{CRB}\left(\theta \right)=\frac{\sigma^{2}}{2L}{\left\{ℜ\left[H⊙{\left(PA^{H}{\hat{R}}^{-1}AP\right)}^{T}\right]\right\}}^{-1}, \end{array}$$

where

(33) $$\begin{array}{cc} \; & H=D^{H}\left(I-A{\left(A^{H}A\right)}^{-1}A^{H}\right)D,\\ \; & D=\left[\begin{array}{ccc} \frac{\partial a(\theta_{1})}{\partial \theta_{1}}, & \ldots , & \frac{\partial a(\theta_{K})}{\partial \theta_{K}} \end{array}\right], \end{array}$$

and $P=\left(1/L\right)SS^{H}$.

Now considering the 2-D L-shaped array case with the angles for the *k*-th source as $\eta_{k}=\left[\theta_{k},\phi_{k}\right]$, under the stochastic signal assupmtion, we can see that $y\left(t\right)∼\mathrm{CN}\left(0,\hat{R}\right)$. The CRB of 2-D DOA estimation for rectangular array has been analyzed in [46], which has some similarity with the L-shaped array. So we can modify it in stochastic assumption to calculate the CRB for L-shaped array.

The stochastic CRB can be calculated as follows:

(34) $$\begin{array}{c} \hat{\mathrm{CRB}}\left(\eta \right)=\frac{1}{L}{\left(F-MG^{-1}M^{T}\right)}^{-1} \end{array}$$

where

(35) $$\begin{array}{c} F=2ℜ\{H^{\prime }⊙\left[{\left(P{\bar{A}}^{H}{\bar{R}}^{-1}\bar{A}P\right)}^{T}⊗\Delta \right]\} \end{array}$$

(36) $$\begin{array}{c} M=2ℜ\{\Xi^{T}\left[\left(D^{\prime H}\Pi_{\bar{A}}^{⊥}\right)⊗\left(P_{2}^{T}{\bar{A}}^{T}{\bar{R}}^{-T}\right)\right]\Lambda^{*}\} \end{array}$$

(37) $$\begin{array}{c} G=2ℜ\left[\Lambda^{H}\left({\bar{R}}^{-T}⊗\Pi_{\bar{A}}^{⊥}\right)\Lambda \right]-\Lambda^{H}\left[{\left(\Pi_{\bar{A}}^{⊥}\right)}^{T}⊗\Pi_{\bar{A}}^{⊥}\right]\Lambda \end{array}$$

where

(38) $$\bar{A}=\sigma^{-\frac{1}{2}}A\left(\phi ,\theta \right),\bar{R}=\sigma^{-1}\tilde{R},$$

(39) $$\Pi_{\bar{A}}^{⊥}=I_{M}-\bar{A}{\left({\bar{A}}^{H}\bar{A}\right)}^{-1}{\bar{A}}^{H}$$

(40) $$H^{\prime }=D^{\prime H}\Pi_{\bar{A}}^{⊥}D^{\prime },$$

(41) $$P_{2}=P⊗\left[1,1\right],\Delta =\left[\begin{array}{cc} 1 & 1\\ 1 & 1 \end{array}\right],$$

(42) $$\begin{array}{c} D^{\prime }=\sigma^{-\frac{1}{2}}\left[\begin{array}{ccc} \frac{\partial a(\phi_{1},\theta_{1})}{\partial \eta_{1}^{T}} & \cdots & \frac{\partial a(\phi_{K},\theta_{K})}{\partial \eta_{K}^{T}} \end{array}\right], \end{array}$$

(43) $$\begin{array}{c} \Lambda =\left[\begin{array}{ccc} \mathrm{vec}{\left(I_{M}\right)}^{T} & \cdots & \mathrm{vec}{\left(I_{M}\right)}^{T} \end{array}\right], \end{array}$$

(44) $$\Xi =\left[\begin{array}{ccc} \mathrm{vec}(e_{1}e_{1}^{T}) & \cdots & \mathrm{vec}(e_{2K}e_{2K}^{T}) \end{array}\right],$$

where $e_{k}$ denotes the *k*-th column of a K×K identity matrix. Note that as the noise variance is equivalent to σI, the CRB can be simplified as follows [46]:

(45) $$\hat{\mathrm{CRB}}\left(\theta ,\phi \right)=\frac{1}{L}F^{-1}.$$

## 6. Numerical Simulations

In this section, we illustrate the performance of the proposed method in comparison with existing approaches as follows:

1. ESPRIT: The classical ESPRIT algorithm [41] is directly applied to the sample covariance matrix $\tilde{R}$ in (4) with the elevation angles and azimuth angles auto-paired.
2. SPA [33]: The SPA is separately applied to the two subarrays to estimate the elevation angles and azimuth angles, and the probable pairing error is neglected in this process.
3. JSR: The JSR framework is implemented as described in Corollary 1 with the regularization parameters λ set as [39].
4. CRB: The average lower bounds for estimation separately implemented in two subarrays is denoted as CRB, which is calculated as (32).
5. CRB+: The lower bounds for joint 2-D DOA estimation based on the whole covariance matrix is represented as CRB+, which is calculated as (45).

The proposed method is implemented by the SDP3 solver [47] if not stated otherwise. ADMM will be terminated if a maximum number of 2000 iterations is reached. The complex amplitude $\left\{s_{k}\left(t\right)\right\}$ is generated randomly from a standard complex normal distribution, and different angles $\eta_{k}=\left\{\phi_{k},\theta_{k}\right\}$ are set for distinct sources. The noise is complex Gaussian white noise with zero mean and the SNR is defined as $10\log_{10}{(∥Y-E∥}_{F}^{2}/{∥E∥}_{F}^{2})$ dB.

### 6.1. Effectiveness and Identifiability

**Figure 2:**
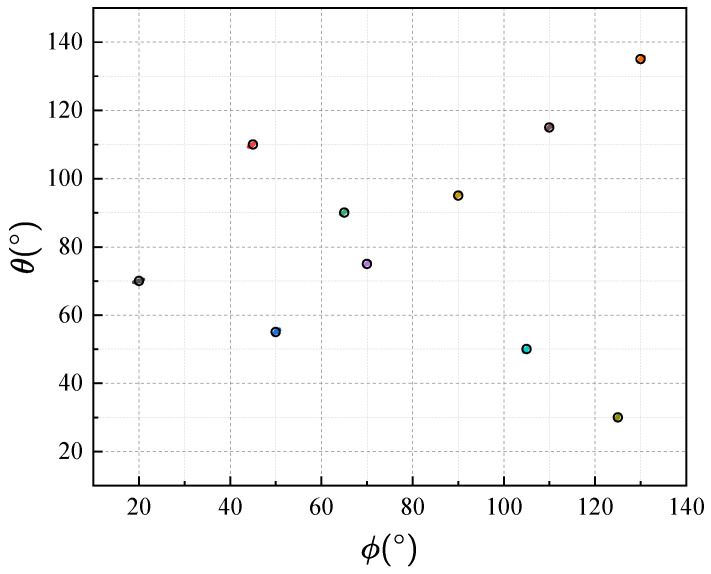
Two-dimensional DOA estimation of ten uncorrelated components (Solid dots of different colors indicate the estimated frequency groups while the black circles indicate the true frequency groups).

In experiment 1, we verify the effectiveness of the proposed method by ADMM solver and attempt to estimate K=10 uncorrelated targets. Moreover, we set L=200, $M_{x}=M_{z}=15$, and SNR=10dB and a number of 200 Monte Carlo runs are carried out under this condition. The estimation results are shown in Figure 2 with the black circles indicating the true frequency groups. It is seen that frequency groups can be effectively estimated and correctly paired using the proposed method.

**Figure 3:**
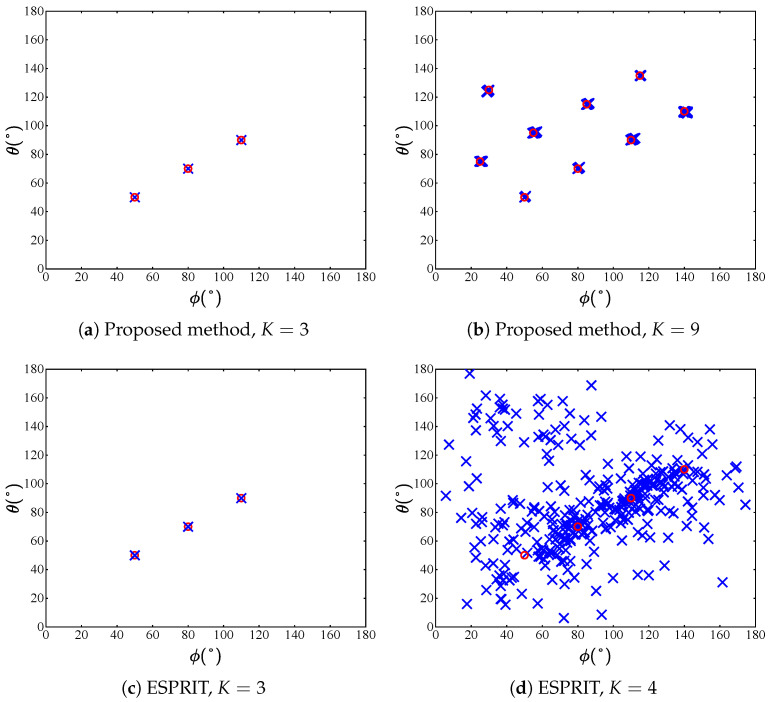
Estimates of the proposed method and ESPRIT with 100 trials, where the number of sensors is set as $M_{x}=4$ and $M_{z}=4$ (red circles and blue crosses denote the real values and the estimates, respectively).

In experiment 2, to validate the superiority of the proposed method in terms of the maximum identifiable targets, we employ $M_{x}=4,M_{z}=4$ sensors and $L=1\times 10^{6}$ snapshots in the high SNR regime as SNR=30dB to obtain a relatively ideal sample covariance matrix. Figure 3 shows the parameter identifiability of the proposed method compared with the ESPRIT algorithm. The simulations are provided to illustrate that the maximum identifiable targets of the proposed method when $M_{x}=M_{z}=4$ is K=9, while that of ESPRIT is K=3. It can be seen that when the number of sources is smaller than sensors in each subarray, the proposed method and ESPRIT both exhibit accurate estimation results, while in the case of more sources, only the proposed method still maintains successful estimation.

### 6.2. Statistic Performance Versus SNR and Snapshots

In experiment 3, the estimation accuracy of the proposed method versus SNR and the number of snapshots is studied. We suppose K=2 uncorrelated sources are received at azimuth angles ϕ={60,90} and elevation angles θ={50,80}. J=200 Monte Carlo trials are carried out, and the root mean square error (RMSE) of the system can be calculated as follows:

(46) $$\begin{array}{c} \mathrm{RMSE}\left(\phi ,\theta \right)=\frac{1}{K}\sqrt{\frac{1}{J}\sum_{j=1}^{J}\sum_{k=1}^{K}{\left({\hat{\phi }}_{jk}-\phi_{k}\right)}^{2}+{\left({\hat{\theta }}_{jk}-\theta_{k}\right)}^{2}}, \end{array}$$

where ${\hat{\phi }}_{jk}$ and ${\hat{\theta }}_{jk}$ denote the azimuth and elevation angles of the estimates of the *k*-th source in the *j*-th trial.

As a few outliers may lower the reliability of the experiments, we set a threshold to measure the success of a single experiment, which means that if there exists any source with estimate error with $\left|{\hat{\theta }}_{jk}-\theta_{k}\right|>30^{\circ }$ or $\left|{\hat{\phi }}_{jk}-\phi_{k}\right|>30^{\circ }$, the experiment will be considered as a failure and it will not be included in the calculation of RMSE.

**Figure 4:**
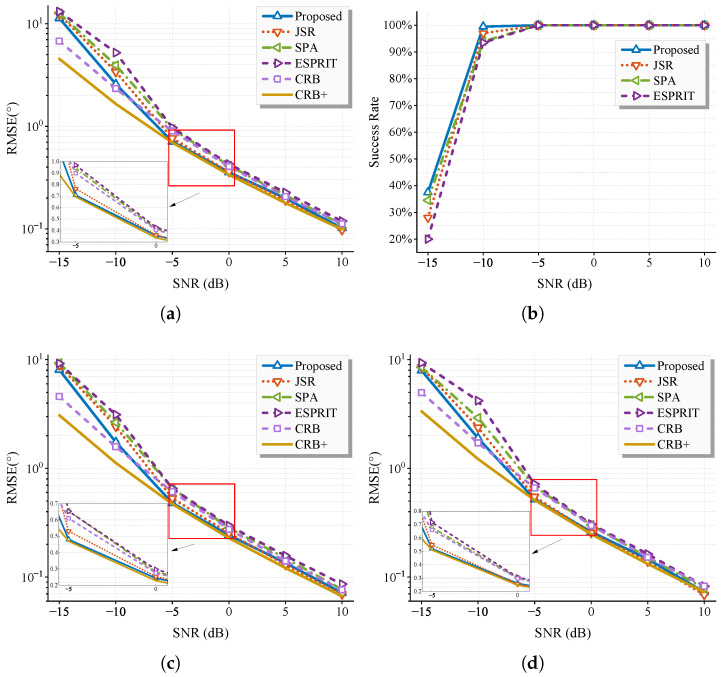
RMSE of estimate results of (**a**) the whole system, (**b**) success rate with threshold set as $30^{\circ }$, (**c**) x-axis, and (**d**) z-axis. Some settings: $M_{x}=6,M_{z}=6$, K=2, L=200, $\theta_{1}\;=\;\left\{60,90\right\}$, and $\theta_{2}\;=\;\left\{50,80\right\}$.

First, we fix L=200, $M_{x}=6$, $M_{z}=6$, and K=2 and SNR varies over the range {−15,−10,…,10} dB. Figure 4 displays the RMSE of the L-shaped array, the success rate and the RMSE of two subarrays. The proposed method demonstrates similar performance with JSR, consistent with theoretical analysis. Consequently, subsequent comparisons focus on the comparison of the proposed method with the other two methods. All algorithms exhibit strong performance in high SNR regimes. The proposed method outperforms others by fully exploiting redundant information embedded within the whole covariance matrix, achieving lower CRB. In moderate/low SNR regimes (SNR≤−5dB), all methods experience RMSE degradation, though the proposed approach demonstrates superior noise robustness. Under extreme low-SNR conditions (−15 dB), while all algorithms fail to provide accurate estimates, the proposed method maintains a marginal performance advantage.

**Figure 5:**
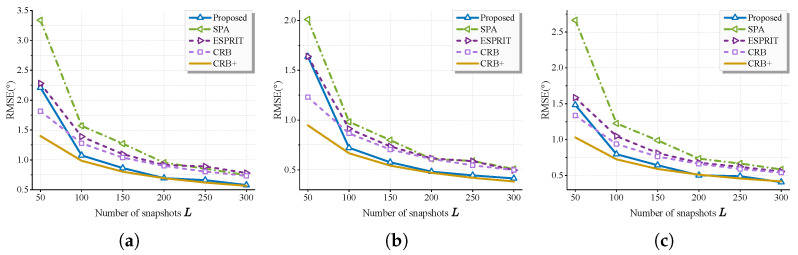
RMSE of estimate results of (**a**) the whole system, (**b**) x-axis, (**c**) z-axis. Some settings: $M_{x}=6,M_{z}=6$, K=2, SNR=5dB, ϕ={60,90}, and θ={50,80}.

With SNR fixed at 5 dB and other parameters unchanged ($M_{x}=6$, $M_{z}=6$, K=2), *L* varies across {50,100,…,300}. Figure 5 presents the RMSE for the L-shaped array and two subarrays, where all methods achieve a 100% success rate. Under moderate SNR, all algorithms exhibit RMSE convergence toward their respective CRB with increasing *L*. The proposed method asymptotically attains the CRB+ at large *L*, demonstrating its equivalence to a large-sample realization of the maximum likelihood estimator, and maintains consistent superiority over the other two methods, attributed to its lower CRB.

### 6.3. Complexity Analysis

Since the computation of the sample covariance matrix is consistent across all methods, the complexity of data preprocessing is not analyzed separately. Except for subspace-based methods, all comparative algorithms consist of two main steps, i.e., optimization and DOA extraction. Therefore, the computational complexity is analyzed in these two parts with the optimization problems all solved via SDPT3 [47] and the DOA estimation performed by either ESPRIT or MD-ESPRIT. Suppose $n_{1}=M^{2}+2M_{x}M_{z}-M,n_{2}=M+M_{x}M_{z}$; the complexity of different methods for 2-D DOA estimation is summarized as Table 1.

It can be seen that the subspace-based method exhibits significantly lower complexity than both SPA and the proposed method, with the complexity of all the algorithms primarily influenced by the number of sensors.

**Figure 6:**
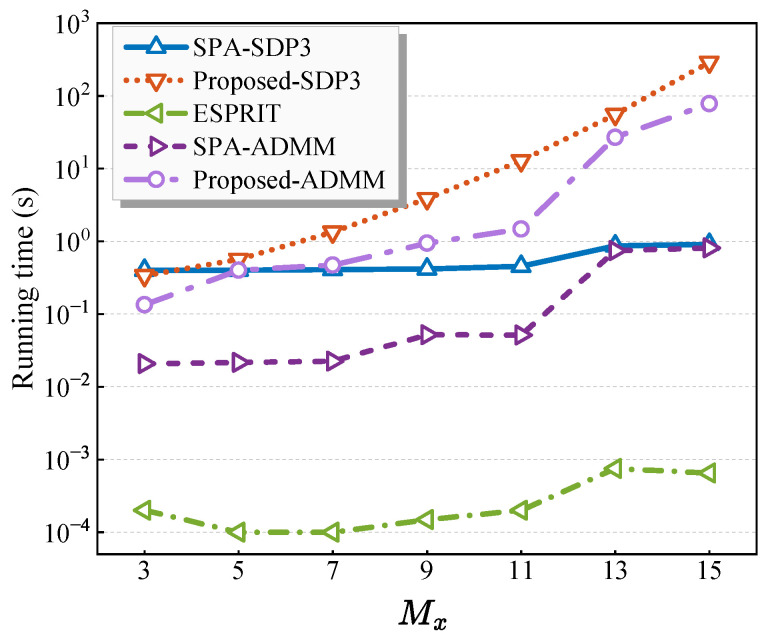
Average running time of algorithms versus the number of sensors.

In addition, this section evaluates the effectiveness of the ADMM algorithm in reducing the complexity of the optimization process through simulation experiments. Figure 6 compares the average running time of the proposed method implemented via the SDP3 solver and ADMM with the number of sensors $M_{x}=\left\{3,5,7,8,11,13,15\right\},M_{z}=M_{x}$, L=200, and SNR=0dB. Computational costs of the ESPRIT and the SPA methods are also exhibited for comparison. Compared to the SDP3 solver, ADMM achieves significant computational savings, consistent with theoretical expectations. Besides, ADMM termination criteria critically govern accuracy–complexity tradeoffs, enabling tunable performance balancing. Although the proposed method exhibits higher complexity than others, the practical complexity gaps between SPA and the proposed method may narrow as SPA here omits pairing operations.

### 6.4. Resolution Performance of Closely Located Targets

Since the 2-D MUSIC method is capable of utilizing all the data in the L-shaped array, experiment 4 compares the spectral performance of the proposed method and 2-D MUSIC with limited snapshots (L=6). K=4 uncorrelated targets impinge on the system with $M_{x}=10$, $M_{z}=10$ sensors at SNR=20dB. Uniform grids span $0^{\circ }$ to $180^{\circ }$ with 1800 points per dimension, and the results are normalized for comparision. Figure 5 depicts the spectra under varying angular separations.

**Figure 7:**
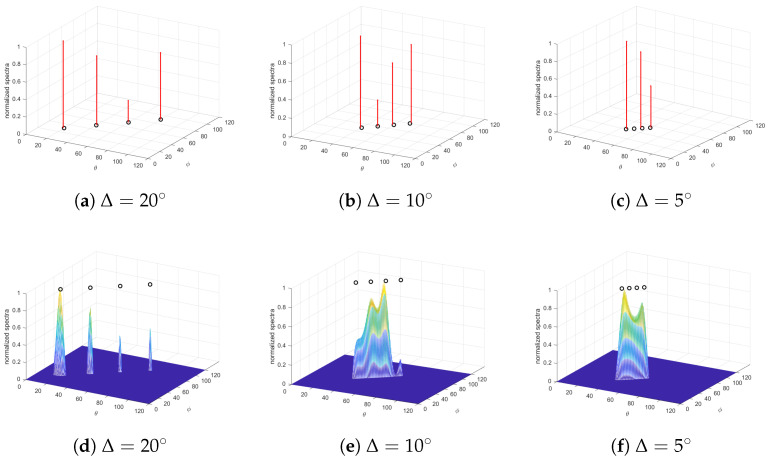
Spectra of the proposed method (the first row) and 2-D MUSIC (the second row) for uncorrelated targets. Simulation parameters: $M_{x}=10,M_{z}=10$, K=4, L=6 and the angular separation is Δ = (**a**,**d**) $20^{\circ }$, (**b**,**e**) $10^{\circ }$, and (**c**,**f**) $5^{\circ }$ at both subarrays.

Both methods demonstrate comparable performance for targets with large angular separation (Figure 7a,d). However, as separation decreases, performance deterioration occurs, manifesting as inaccurate target identification or partial estimation failure. It is attributed to heightened correlation between closely spaced targets. Crucially, the proposed method maintains superiority over 2-D MUSIC at moderate separations (Figure 7b,e). Additionally, the resolution of MUSIC depends on grid density, inducing a larger computational load for better performance.

## 7. Conclusions

This paper presents a 2-D DOA estimation method for L-shaped arrays via multidimensional structured covariance matrix completion. By exploiting the intrinsic connection between the MLT structure and the ideal covariance matrix, we formulate an SDP problem incorporating joint sparsity and low-rank constraints. Unlike existing sparse methods that rely solely on cross-correlation matrices, the proposed method leverages the redundant information embedded in the whole covariance matrix to enhance estimation accuracy. An efficient ADMM implementation reduces computational complexity. Simulation results validate that the proposed method is capable of identifying more targets than current methods and achieving higher estimation accuracy attributed to its lower CRB. Besides, the proposed method exhibits better resolution for closely spaced targets with limited snapshots compared to 2-D MUSIC. Although the ADMM algorithm is capable of lowering the computational complexity of the proposed method, its complexity is still higher than that of most current methods. In future research, we will explore methods to further reduce the complexity and study the necessary and sufficient condition for the unique Vandermonde decomposition of an MLT matrix. 

## Figures and Tables

**Table 1:** Complexity of different methods for 2-D DOA estimation.

Method	Optimization	DOA Estimation
ESPRIT	-	$O(M^{3}+K^{2}M+K^{3})$
SPA	$O(M_{x}^{6.5}+M_{z}^{6.5})$	$O(M_{x}^{3}+M_{z}^{3}+K^{2}\left(M_{x}+M_{z}\right)+K^{3})$
Proposed method	$O(n_{1}^{2}n_{2}^{2.5})$	$O({\left(M_{x}M_{z}\right)}^{3}+K^{2}M_{x}M_{z}+K^{3})$

## Data Availability

Data are contained within the article.

## Appendix A. Proof of Proposition 1

For brevity, we substitute the MLT matrix $T_{N}$ with T in this Appendix.

**Lemma** **A1.**
*The following holds for arbitrary vectors ${\left\{a_{i}\right\}}_{i=1}^{n}$:*

(A1)
$$\begin{array}{c} \left(a_{1}\;⊗\;\cdots \;⊗\;a_{n}\right){\left(a_{1}\;⊗\;\cdots \;⊗\;a_{n}\right)}^{H}\;=\;\left(a_{1}a_{1}^{H}\;⊗\;\cdots \;⊗\;a_{n}a_{n}^{H}\right). \end{array}$$

**Proof** **of** **Lemma** **A1.** Let $a_{i}a_{i}^{H}=A_{i}$ and $B_{i}=a_{1}⊗\cdots ⊗a_{i}$ for ease of notation. We make use of the basic properties of the Kronecker product as follows:

(A2) $$\left(AB\right)⊗\left(CD\right)=\mathrm{AC}⊗\mathrm{BD},\;{\left(A⊗B\right)}^{H}=\left(A^{H}⊗B^{H}\right)$$

According to properties above, it is intuitive that the lemma holds for n=1,2, and the left equation of (A1) can easily be simplified as follows:

(A3) $$\mathrm{left}\;\mathrm{side}=\left(a_{1}⊗\cdots ⊗a_{n}\right)\left(a_{1}^{H}⊗\cdots ⊗a_{n}^{H}\right).$$

Then suppose the lemma holds for n=m(m≥2), written as follows:

(A4) $$B_{m}B_{m}^{H}=A_{1}⊗\cdots ⊗A_{m},$$

which is sufficient to prove that the lemma also holds for n=m+1 as follows:

(A5) $$\begin{array}{cc} B_{m+1}B_{m+1}^{H} & =\left(B_{m}⊗a_{m+1}\right){\left(B_{m}⊗a_{m+1}\right)}^{H}\\ \; & =(a_{1}a_{1}^{H}⊗\cdots ⊗a_{m+1}a_{m+1}^{H}). \end{array}$$

According to mathematical induction method, the lemma is proved. □

We can prove Proposition 1 as follows. Without loss of generality, assume that n≥m in $R_{mn}$ and let $T_{q}\in C^{N_{q}\times N_{q}}$ denotes a submatrix of T as follows:

(A6) $$\begin{array}{cc} T_{q} & =T\left(1:N_{q},1:N_{q}\right)=\sum_{k=1}^{r}p_{k}\left(a_{qk}⊗\cdots ⊗a_{1k}\right){\left(a_{qk}⊗\cdots ⊗a_{1k}\right)}^{H}\\ \; & =\sum_{k=1}^{r}p_{k}\left(a_{qk}a_{qk}^{H}⊗\cdots ⊗a_{1k}a_{1k}^{H}\right), \end{array}$$

where $\mathrm{rank}\left(T\right)=r<\min{\left\{M_{n}\right\}}_{n=1}^{N}$, then define $Q_{qk}$ as follows:

(A7) $$Q_{qk}=\left(a_{qk}a_{qk}^{H}⊗\cdots ⊗a_{1k}a_{1k}^{H}\right)\in C^{N_{q}\times N_{q}},\;q\leq N,$$

where $a_{nk}=\left[1,\phi_{nk},\cdots ,\phi_{nk}^{M_{n}-1}\right]$, $\phi_{nk}=e^{i2\pi f_{nk}}$.

It is intuitive that $Q_{nk}$ defined as (A7) can be calculated recursively as follows:

(A8) $$\begin{array}{cc} Q_{nk} & \;=\;\left(a_{nk}a_{nk}^{H}⊗Q_{(n-1)k}\right)\;=\;\left[\begin{array}{cccc} 1Q_{(n-1)k} & \;\phi_{nk}^{-1}Q_{(n-1)k} & \;\cdots & \;\phi_{nk}^{1-M_{n}}Q_{(n-1)k}\\ \phi_{nk}Q_{(n-1)k} & \;1Q_{(n-1)k} & \;\cdots & \;\phi_{nk}^{2-M_{n}}Q_{(n-1)k}\\ \vdots & \;\vdots & \;\ddots & \;\vdots \\ \phi_{nk}^{M_{n}-1}Q_{(n-1)k} & \;\phi_{nk}^{M_{n}-2}Q_{(n-1)k} & \;\cdots & \;1Q_{(n-1)k} \end{array}\right]. \end{array}$$

Then it is clear that the first $N_{n-1}$ rows of $Q_{nk}$ are equal to $a_{nk}^{H}⊗Q_{(n-1)k}\in C^{N_{n-1}\times M_{n}}$. Therefore, we can get $a_{nk}^{H}$ by taking interval values from the first row of $Q_{nk}$ as follows:

(A9) $$a_{nk}^{H}=Q_{nk}\left(1,1:N_{n-1}:N_{n}\right).$$

In addition, under the assumption that n≥m, $Q_{mk}=Q_{nk}\left(1:N_{m},1:N_{m}\right)$, then the following is shown:

(A10) $$a_{mk}=Q_{nk}\left(1:N_{m-1}:N_{m},1\right).$$

According to the results above, the following holds:

(A11) $$\begin{array}{cc} R_{mn} & \;=\;\sum_{k=1}^{r}p_{k}a_{mk}a_{nk}^{H}\;=\;\sum_{k=1}^{r}p_{k}Q_{nk}\left(1:N_{m-1}:N_{m},1\right)Q_{nk}\left(1,1:N_{n-1}:N_{n}\right)\\ \; & \;=\;\sum_{k=1}^{r}p_{k}Q_{Nk}\left(1:N_{m-1}:N_{m},1\right)Q_{Nk}\left(1,1:N_{n-1}:N_{n}\right). \end{array}$$

Let $W_{k}\;=\;a_{Nk}⊗\cdots ⊗a_{1k}$, and the following holds:

(A12) $$\begin{array}{c} Q_{Nk}\left(p,1\right)=W_{k}\left(p\right),\;Q_{Nk}\left(1,q\right)=W_{k}{\left(q\right)}^{*}, \end{array}$$

then for elements of T, the following always holds:

(A13) $$\begin{array}{cc} T(p,q) & =\sum_{k=1}^{r}p_{k}W_{k}\left(p\right)W_{k}{\left(q\right)}^{*}=\sum_{k=1}^{r}p_{k}Q_{Nk}\left(p,1\right)Q_{Nk}\left(1,q\right). \end{array}$$

And the following holds:

(A14) $$\begin{array}{c} R_{mn}=T\left(1:N_{m-1}:N_{m},1:N_{n-1}:N_{n}\right). \end{array}$$

Moreover, the following always holds:

(A15) $$R_{mn}=R_{nm}^{H}.$$

Therefore, Proposition 1 is proved.

## Appendix B. Proof of Theorem 2

Suppose that $W={\left(R+\epsilon I\right)}^{-1}$, which can be considered the inverse of ideal covariance matrix, then according to Woodbury matrix inversion lemma [48], the following holds:

(A16) $$\begin{array}{c} W=\frac{1}{\epsilon }\left[I-A{\left(\epsilon P^{-1}+A^{H}A\right)}^{-1}A^{H}\right], \end{array}$$

where $A={\left[\begin{array}{c} A_{x}^{T},A_{z}^{T} \end{array}\right]}^{T}$ is manifold matrix of R, then the diagonal block matrices $W_{x/z}$ and off-diagonal block matrices $W_{xz}$ can be represented as follows:

(A17) $$\begin{array}{cc} W_{x} & =\frac{1}{\epsilon }\left[I_{M_{1}}-A_{x}{\left(\epsilon P^{-1}+A_{x}^{H}A_{x}\right)}^{-1}A_{x}^{H}\right],\\ W_{z} & =\frac{1}{\epsilon }\left[I_{M_{2}-1}-A_{z}{\left(\epsilon P^{-1}+A_{z}^{H}A_{z}\right)}^{-1}A_{z}^{H}\right],\\ W_{xz} & =-\frac{1}{\epsilon }A_{x}{\left(\epsilon P^{-1}+A^{H}A\right)}^{-1}A_{z}^{H}. \end{array}$$

Let $Q=\epsilon P^{-1}+A^{H}A$, then the following holds:

(A18) $$\begin{array}{c} Q=\epsilon P^{-1}+A_{x}^{H}A_{x}+A_{z}^{H}A_{z}=\epsilon P^{-1}+MI_{K} \end{array}$$

for there always exists the following: $a_{xk}^{H}a_{xk}=M_{1}$, $a_{zk}^{H}a_{zk}=M_{2}-1$.

Suppose that $\delta_{xk}=Q^{-\frac{1}{2}}A_{x}^{H}a_{xk}$, then we have the following:

(A19) $$\begin{array}{cc} w_{xk} & =a_{xk}^{H}W_{x}a_{xk}=\frac{1}{\epsilon }a_{xk}^{H}\left(I-A_{x}Q^{-1}A_{x}^{H}\right)a_{xk}\\ \; & =\frac{1}{\epsilon }(M_{1}-∥\delta_{xk}{∥}_{2}^{2}), \end{array}$$

(A20) $$\begin{array}{cc} w_{xzk} & =a_{xk}^{H}W_{xz}a_{zk}=-\frac{1}{\epsilon }a_{xk}^{H}A_{x}Q^{-1}A_{z}^{H}a_{zk}\\ \; & =-\frac{1}{\epsilon }\left(\delta_{xk}^{H}\delta_{zk}\right), \end{array}$$

and $w_{zk}$ can be calculated similar to $w_{xk}$. Subsequently, $w_{k}$ can be computed as follows:

(A21) $$\begin{array}{cc} \; & w_{k}=[\frac{1}{M}(a_{xk}^{H}W_{x}a_{xk}+a_{zk}^{H}W_{z}a_{zk}\\ \; & +a_{xk}^{H}W_{xz}a_{zk}+a_{zk}^{H}W_{xz}^{H}a_{xk}{)]}^{-\frac{1}{2}}\\ \; & =\sqrt{M\epsilon }[(M_{1}-∥\delta_{xk}{∥}_{2}^{2})+(M_{2}-1-∥\delta_{zk}{∥}_{2}^{2})\\ & -\delta_{xk}^{H}\delta_{zk}-\delta_{zk}^{H}\delta_{xk}{]}^{-\frac{1}{2}}\\ \; & =\sqrt{M\epsilon }(M-∥\delta_{xk}+\delta_{zk}{{∥}_{2}^{2})}^{-\frac{1}{2}}\\ & =\sqrt{\epsilon }(1-\frac{1}{M}∥Q^{-\frac{1}{2}}\left(A_{x}^{H}a_{xk}+A_{z}^{H}a_{zk}\right){{∥}_{2}^{2})}^{-\frac{1}{2}}. \end{array}$$

Then Theorem 2 is proved.
